# The effectiveness of dry needling for plantar fasciitis: a systematic review and meta-analysis

**DOI:** 10.3389/fneur.2024.1520585

**Published:** 2024-12-18

**Authors:** Aiguo Yang, Rong Lin, Mingwei Xia, Hao Su, Ying He

**Affiliations:** ^1^Department of Traditional Chinese Medicine, Affiliated Hospital and Clinical Medical College of Chengdu University, Chengdu, China; ^2^Department of Rehabilitation Medicine, The Traditional Chinese Medicine Hospital of Longquanyi, Chengdu, China; ^3^Department of Rehabilitation Medicine, Bazhong Central Hospital, Bazhong, China; ^4^Department of Rehabilitation Medicine, Affiliated Hospital and Clinical Medical College of Chengdu University, Chengdu, China

**Keywords:** dry needling, plantar fasciitis, pain, systematic review, meta-analysis

## Abstract

**Objective:**

To evaluate the effectiveness of dry needling (DN) on pain and functional outcomes in patients with plantar fasciitis (PF).

**Methods:**

PubMed, Embase, the Cochrane Library, EBSCO, web of science, physiotherapy Evidence Database (PEDro) were searched for randomized control trials (RCTs) evaluating the effectiveness of dry needling on plantar fasciitis. Article screening, data extraction and risk-of-bias evaluation were independently performed by two reviewers. Meta-analysis was conducted based on different control methods and assessment time using RevMan 5.3 software.

**Results:**

A total of 12 RCTs involving 781 patients were included in the systemic review and meta-analysis. The comparison of DN + routine treatments vs. routine treatments alone demonstrated that PF patients receiving DN have significantly lower scores in Visual Analog Scale / Numerical Pain Rating Scale (VAS/NPRS) [95%CI (−2.12, −1.76), *p* < 0.0001], and the scores of Foot Function Index (FFI) [95%CI (−12.57, −3.58), *p* = 0.004]. In the comparison of DN vs. other treatments, results showed that DN significantly lowered the scores of FFI [95%CI (−6.55, −1.09), *p* = 0.006]. However, there was no significant difference in pain improvement between DN and other treatments [95%CI (−0.66, 0.06), *p* = 0.10]. In the meta-analysis based on different assessment time, results showed that there was significant difference in the scores of VAS/NPRS within 1 month, at 1 month and at over 1 month. But there was no significant difference in the scores of FFI within 1 month, while at 1 month and at over 1 month, the scores of FFI were lowered in patients receiving DN, and the difference were statistically significant.

**Conclusion:**

Dry needling is effective in relieving pain and restoring function in patients with plantar fasciitis. Furthermore, dry needling may take at least 1 month to take effects in patients with plantar fasciitis. More multi-center RCTs with high-quality, large sample size are needed to further conform our conclusion.

## 1 Introduction

Plantar fasciitis (PF) is the most common cause of inferior heel pain. The lifetime prevalence of PF has reached 10% in general population, and it is even higher among athletes, military members, obese people, and those with flatfoot or diabetes mellitus (1, 2). PF is characterized by pain exacerbated with the first walking in the morning or after a long period of rest (3). PF can lead to difficulties in standing, walking, sleeping, and in severe cases, to partial loss of walking function (4). The current treatments for PF often involve the use of physical therapy, orthotic devices, nonsteroidal anti-inflammatory drugs, local steroid injection, splinting and walking cast (5). However, PF has long disease course, slow recovery rate and susceptibility to recurrence (6), stirring up great interest among researchers in the study of the treatment for PF.

Myofascial trigger points (MTrPs) are palpable and hyperirritable nodules located in the taut bands of skeletal muscles (7). In the review by Vincenzo and colleagues (8), it was shown that MTrPs had abnormally contracted sarcomeres and formed contractile knots along the muscle fibers. The gaps between the contraction knots reveal the presence of microcracks and clefts in the endomysium. In addition, MTrPs contraction knots are surrounded by high concentrations of glycosaminoglycans, which are very hygroscopicous molecules, resulting in the trapping of toxic chemicals in the extracellular matrix of muscle tissue. Recently, dry needling (DN) based on MTrPs theory has been proved effective in relieving pain caused by muscle strain, including PF. Previously, five systematic reviews (SRs) (9–13) have indicated that DN exerted favorable effects on PF, however, they also addressed the insufficiency of evidence due to the poor design of the randomized controlled trials (RCTs) analyzed. Furthermore, high heterogeneity has been demonstrated across RCTs regarding to their study design, particularly, the controls. Moreover, when the treatment takes effects, a question that many clinicians are frequently asked about, is still uncertain. In addition, the safety issue of DN is a great concern to many. Therefore, the primary purpose of the current SR was to evaluate the effectiveness of DN on pain and functional outcomes in patients with PF, by carrying out meta-analysis on different control methods, and the second purpose was to investigate the effectiveness of DN on PF at different assessment time. Safety issues of DN treating PF were also discussed.

## 2 Methods

This systematic review and meta-analysis was conducted in accordance with Preferred Reporting Items for Systematic Reviews and Meta-Analyses (PRISMA) (14).

### 2.1 .Search strategy

PubMed, Embase, the Cochrane Library, EBSCO, web of science, physiotherapy Evidence Database (PEDro) were searched from their inception to December 2022. Subject headings and free terms of plantar fasciitis and dry needling were used in combine in the search. The search was limited to RCTs without language restriction. Additionally, the reference list of the identified articles and relevant review were manually searched for more references.

### 2.2 Inclusion and exclusion criteria

Study was included if (1) it was an RCT; (2) the participants were diagnosed with plantar fasciitis. The diagnostic criteria of PF were based on the clinical guidelines linked to the International Classification of Function, Disability and Health from the Orthopedic Section of the American Physical Therapy Association; (3) dry needling was used as the intervention. Study was excluded if full text cannot be obtained, or no available data was presented in the RCT.

### 2.3 Literature screening and data extraction

Two authors independently screened all titles and abstracts, and any disagreements were then resolved by a third author. Studies that satisfied the inclusion and exclusion criteria were retrieved for full-text assessment. Data extracted included basic information (authors, date of publication), subject information (age, gender, and sample size), intervention regimen (frequency and treatment sites of DN), risk of bias, outcome measures, and the follow-up time. The outcomes were extracted in the form of Mean ± standard deviation. Incomplete data were further researched by contacting the author.

### 2.4 Quality assessment

Methodologic Quality Criteria List, which was adapted from Cochrane handbook of reviews of interventions and recommended by the updated method guide for systematic reviews in the Cochrane Back and Neck Group, was applied by two reviewers to evaluate the validity of the included studies independently (15, 16). The evaluation outcomes of each research were presented as Yes, No or Unsure (if there were any unsatisfied results or major deficiencies), which represented Low Risk of Bias, High Risk of Bias and Uncertain Risk of Bias, respectively.

### 2.5 Data synthesis and statistical analysis

Pain relief measured by VAS or NPRS (Visual Analog Scale, Numerical Pain Rating Scale) and functional improvement measured by FFI (foot functional index) were used to evaluate the effectiveness of DN. Lower scores of VAS/NPRS and FFI indicate less severe pain and higher foot function, respectively. The scores of VAS and NPRS were synthesized, considering the similarity in the mechanism of the two scales (17). For different purposes, meta-analysis was conducted based on different outcomes (pain and foot function), control methods (DN vs. other treatments, DN + routine treatment vs. routine treatment alone), and assessment time (within 1 month, at 1 month and over 1 month).

Data were analyzed using RevMan 5.3 software which was provided by Cochrane Collaboration. Firstly, chi-square test was applied to examine the heterogeneity of the included studies, if *p*>0.10 and *I^2^*<50%, the studies were considered homogenous and a fixed-effect model was used. On the contrary, if *p*<0.10 and *I^2^*>50%, heterogeneity of the studies were considered high and random effect models was used (18). The effect sizes were measured using mean difference (MD) and 95% confidence interval (CI). if *p* ≤ 0.05, the difference were considered statistically significant; if *p*>0.05, the difference was considered not statistically significant.

## 3 Results

### 3.1 Study selection

**Figure 1:**
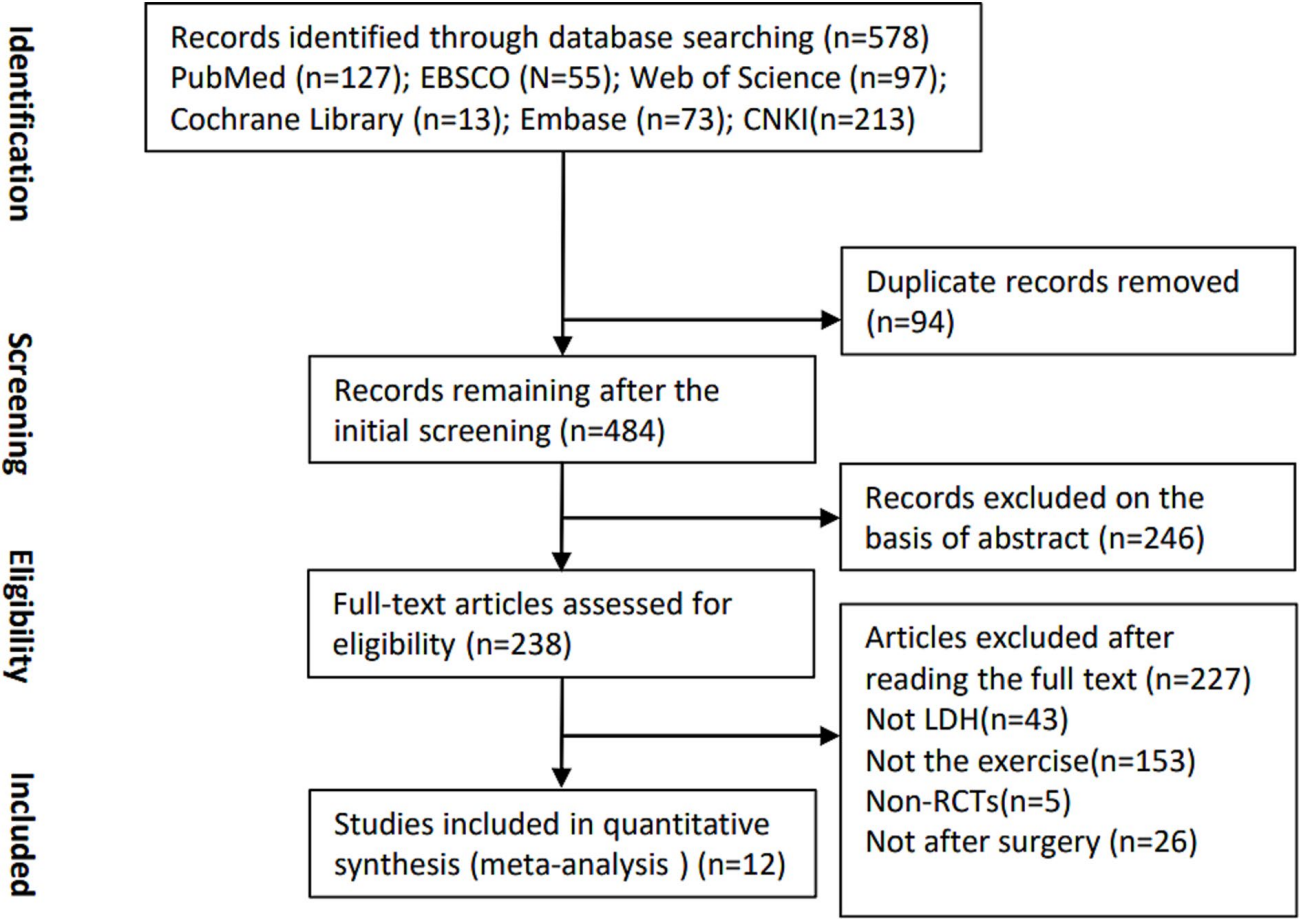
Eligibility of studies for inclusion in meta-analysis.

A total of 238 studies were retrieved from the mentioned databases. After a strict screening process based on the inclusion and exclusion criteria, 12 studies involving 781 participants were eligible and included in current SR and meta-analysis (Figure 1) (19–30).

### 3.2 Study characteristics

The study characteristics of the included RCTs were summarized and presented in Table 1. The publication dates of the included studies ranged from 2014 to 2022. The sample size varied from 20 to 111. Both acute and chronic cases were involved. Adverse events of DN were reported in 6 studies, including pain, bleeding, hematomas and bruising, all of which were self-limiting and easy to be managed. As for the selection of treatment sites, 3 studies used both plantar and gastrocnemius trigger points, 5 studies used only plantar trigger points, 2 study used only gastrocnemius trigger points, 1 study used gastrocnemius and soleus trigger points, and 1 study used soleus, gastrocnemius, quadratus plantae, flexor digitorum brevis and abductor hallucis trigger points according to the degree of pain. The frequency of treatment ranged from 1 to 2 times a week, and the patients received at least 3 sessions of treatment in total.

**Table 1:** Participant characteristics of studies included in this systematic review and meta-analysis.

Study (Design and country)	*n*(M/F)	Age (Intervention group) (Control group)	Intervention group	Control group	Outcome measure	Evaluation time	PEDro scores	Dry needling site	Adverse reactions	Frequency and sessions
Eftekharsadat et al. (23) (RCT, Iran)	20 (10/10)	50.3 ± 9.0 50.9 ± 8.9	DN + massage + Stretching + diclofenac sodium + orthostatic plantar pad	massage + Stretching + diclofenac sodium + orthostatic plantar pad	VAS, ROMPE, ROMDF, FFI	4 weeks, 8 weeks	7/10	MTPs, especially gastrocnemius muscle	Not mentioned	1 session per week for 4 consecutive weeks
Uygur et al. (28) (RCT, Turkey )	98 (33/65)	49.6 ± 11.7 49.9 ± 12.3	DN + drugs + stretching	corticosteroid injection + drugs + stretching	FFI	3 weeks, 6 months	7/10	Plantar MTPs	Pain and/or bleeding	Twice a week for 5 sessions
Dunning et al. (22) (RCT, Spain)	111 (64/47)	39.1 ± 10.4 42.6 ± 11.6	DN + manual therapy + exercise + ultrasound	manual therapy + exercise + ultrasound	FFI, NPRS, LEFS	1 weeks, 4 weeks, 12 weeks	8/10	Plantar and gastrocnemius muscle MTPs	Pain and/or ecchymosis	1–2 times per week for 4 weeks
Rahbar et al. (26) (RCT, Iran)	72 (18/54)	45.08 ± 9.61 43.22 ± 9.20	DN + stretching + orthostatic plantar pad	ESWT + stretching + orthostatic plantar pad	VAS, FFI	4 weeks, 8 weeks	7/10	Plantar MTPs	Pain	1 session per week for 4 consecutive weeks
Rastegar et al. (27) (RCT, Iran)	66 (28/38)	39.84 ± 7.96 42.03 ± 10.30	DN	methylprednisolone acetate injection	VAS	3 weeks, 6 weeks, 12 weeks, 6 months, 12 months	7/10	Plantar MTPs	Not mentioned	Not mentioned
Xie et al. (30) (RCT, China)	48 (26/22)	46.0 ± 10.0 45.6 ± 9.2	DN + stretching	stretching	NPRS, AOFAS, PCS, MCS	4 weeks, 12 weeks	6/10	Gastrocnemius muscle MTPs	Not mentioned	1 session per week for 3 consecutive weeks
Cotchett et al. (21) (RCT, Australia)	84 (44/40)	54.4 ± 12.4 57.8 ± 12.0	DN	sham trigger point dry needling	VAS, FHSQ	2 weeks, 4 weeks, 6 weeks, 12 weeks	8/10	Plantar and gastrocnemius muscle MTPs	Pain and/or ecchymosis	1 session per week for 6 consecutive weeks
El Mallah et al. (24) (RCT, Egypt)	30 (9/21)	45 ± 9 43 ± 10	DN + paracetamol	PRP + paracetamol	FFI	6 weeks, 12 weeks	6/10	Plantar and gastrocnemius muscle MTPs	None	1 session per week for 6 consecutive weeks
Wheeler et al. (29) (RCT, United Kingdom)	90 (30/60)	48.5 ± 9.0 50.4 ± 8.9	DN + autologous blood injection + ultrasound+ exercise	DN + ultrasound +exercise	FFI, NRS, FAAM, MOXFQ, PROMs	2 weeks, 6 weeks, 3 months, and 6 months	8/10	Plantar MTPs	Not mentioned	Not mentioned
Moosaei Saein et al. (25) (RCT, Iran)	20 (0/20)	51.40 ± 5.46 49.40 ± 4.99	DN	none	VAS, ROMDF	4 weeks, 8 weeks	6/10	Gastrocnemius and soleus muscles MTPs	Not mentioned	1 session per week for 4 consecutive weeks
F. Bagcier and Yilmaz (20) (RCT, Turkey)	40 (11/29)	40.1 ± 11.89 47.15 ± 10.82	DN + ESWT+ stretching exercises	ESWT+ stretching exercises	VAS, PPT	1 month	6/10	Gastrocnemius muscle MTPs	Pain	3 sessions with 1-week intervals
Al-Boloushi et al. (19) (RCT, Spain)	102 (30/72)	49.5 ± 8.9 48.1 ± 8.8	DN+ stretching	PNE+ stretching	VAS, FHSQ	4 weeks, 8 weeks, 12 weeks, 26 weeks and 52 weeks	6/10	Soleus, gastrocnemius, quadratus plantae, flexor digitorum brevis and abductor hallucis MTPs	Haematomas	1 session per week for 4 consecutive weeks

M, male; F, female; PEDro, physiotherapy evidence database; RCT, randomized Controlled Trial; DN, dry needling; VAS, visual analog scale; ROMPE, range of motion of plantar extension; ROMDF, range of motion of ankle joint in dorsiflexion; FFI, Foot function index; MTPs, myofascial trigger points; NPRS, Numeric Pain Rating Scale; LEFS, Functional Scale Lower Extremity; ESWT, extracorporeal shockwave; AOFAS, American Orthopedic Foot and Ankle Society Hindfoot Score; PCS, physical component summary; MCS, mental component summary; FHSQ, Foot Health Status Questionnaire; PRP, Platelet-rich plasma; NRS, Numeric Rating Scale; FAAM, Foot and Ankle Ability Measure; MOXFQ, Manchester-Oxford Foot Questionnaire; PROMs, patient-reported outcome measures; PPT, pressure pain threshold; PNE, percutaneous needle electrolysis.

### 3.3 Risk of bias within studies

**Figure 2:**
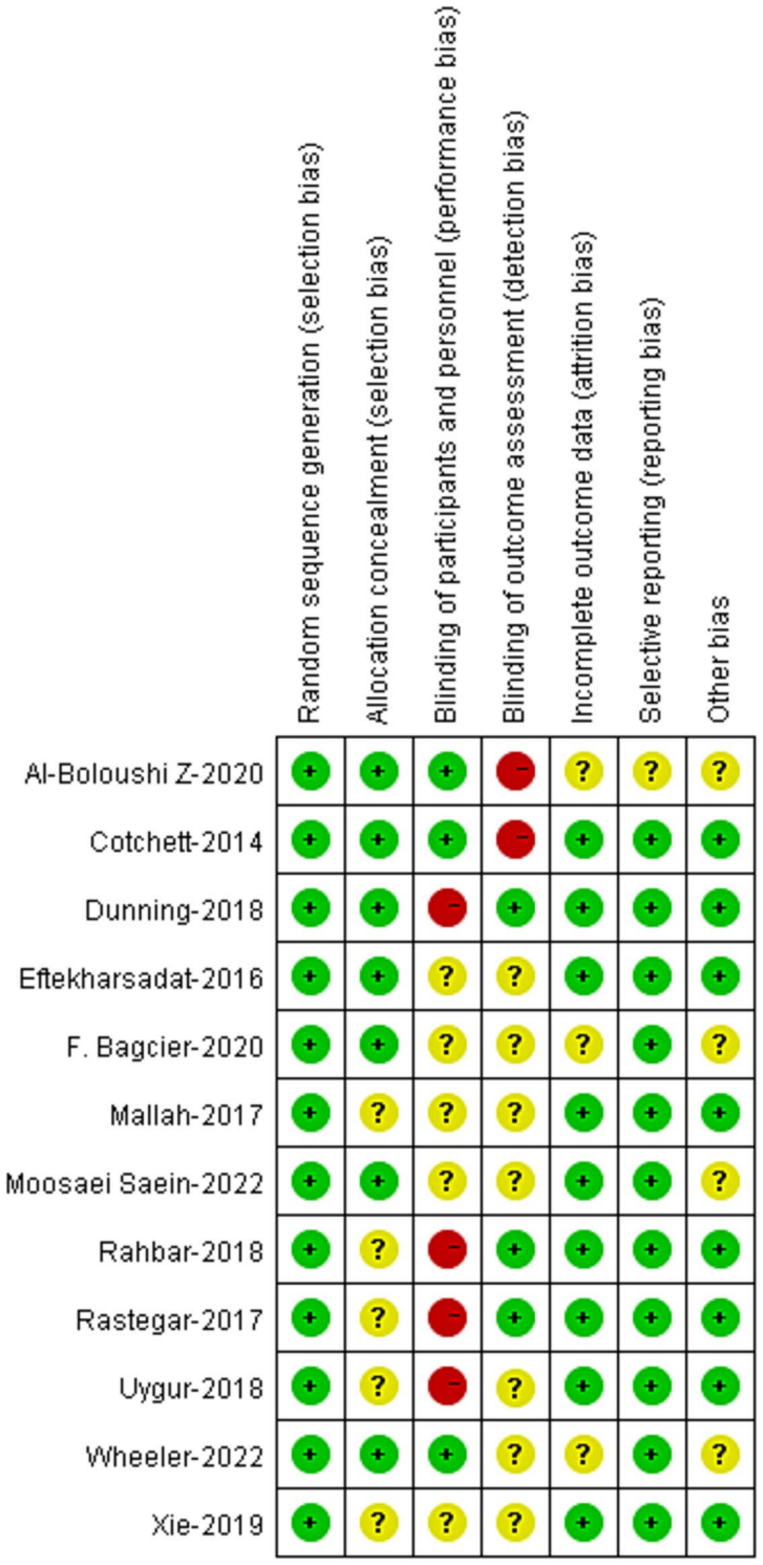
Risk of bias analysis of included studies.

The risk-of-bias of the 12 RCTs were demonstrated in Figure 2. The risk of bias in random allocation were low in the studies. Nevertheless, the risks of bias in allocation concealment remained unclear in 5 studies. Blinding to patients was applied in 3 studies, and blinding to assessors was applied in 3 studies. Other risks of bias were low in these studies.

### 3.4 Meta-analysis based on different control methods

#### 3.4.1 Pain

**Figure 3:**
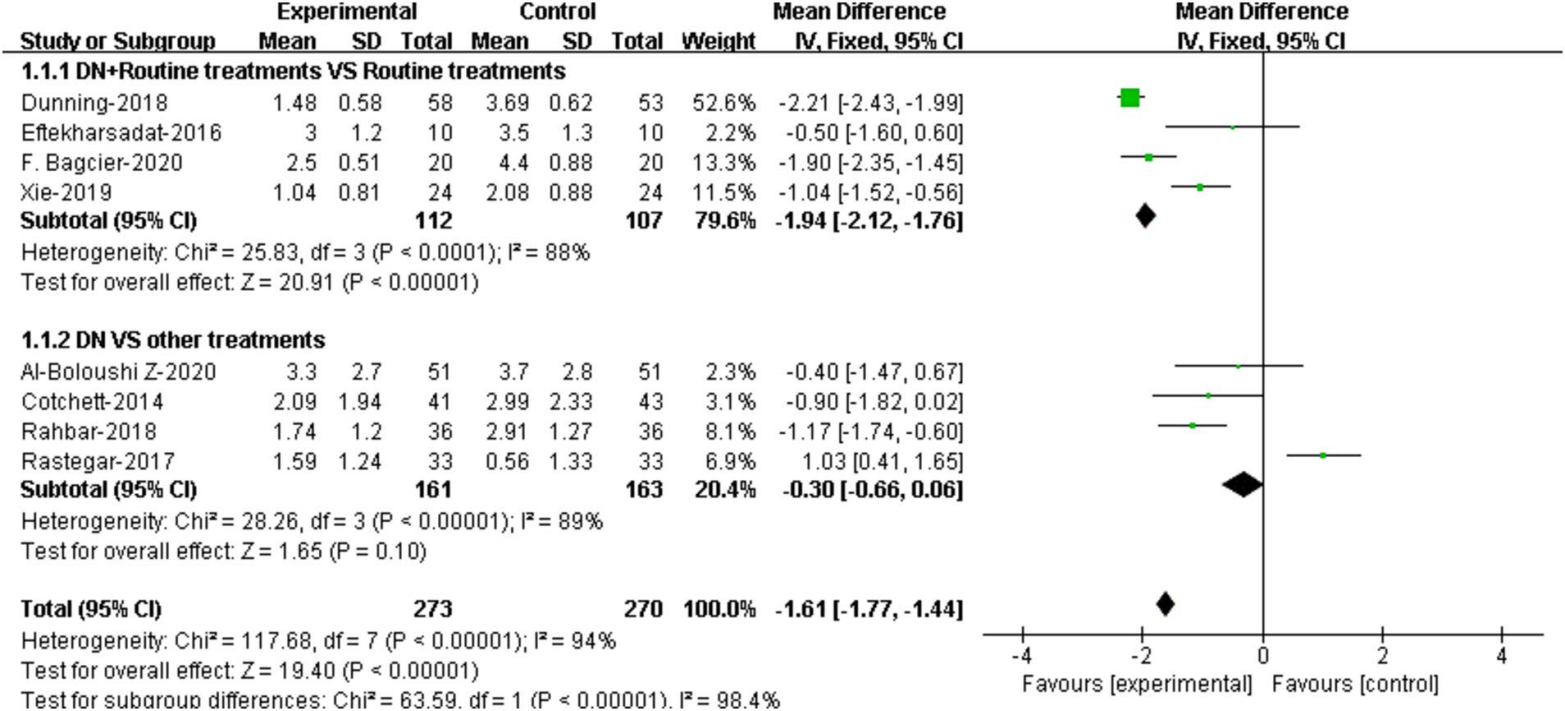
Forest plot illustrating the comparison of effectiveness on pain between DN + routine treatments vs. routine treatments alone, and between DN vs. other treatments.

4 RCTs compared the effectiveness of DN + routine treatment vs. routine treatment alone on pain relieving (20, 22, 23, 30). Routine treatments were defined by the RCTs as muscle massage, stretching, exercise, and ultrasound therapy, etc. Meta-analysis demonstrated that patients who received DN + routine treatment had lower VAS/NPRS scores compared to those who received routine treatment alone, and the difference were statistically significant [95%CI (−2.12, −1.76), *p* < 0.0001] (Figure 3).

The comparison of effectiveness of DN vs. other treatments on pain was reported in 4 RCTs (19, 21, 26, 27). Results showed that there was no significant difference in VAS/NPRS scores between DN group and other treatment group [95%CI (−0.66, 0.06), *p* = 0.10] (Figure 3).

#### 3.4.2 Functional improvement

**Figure 4:**
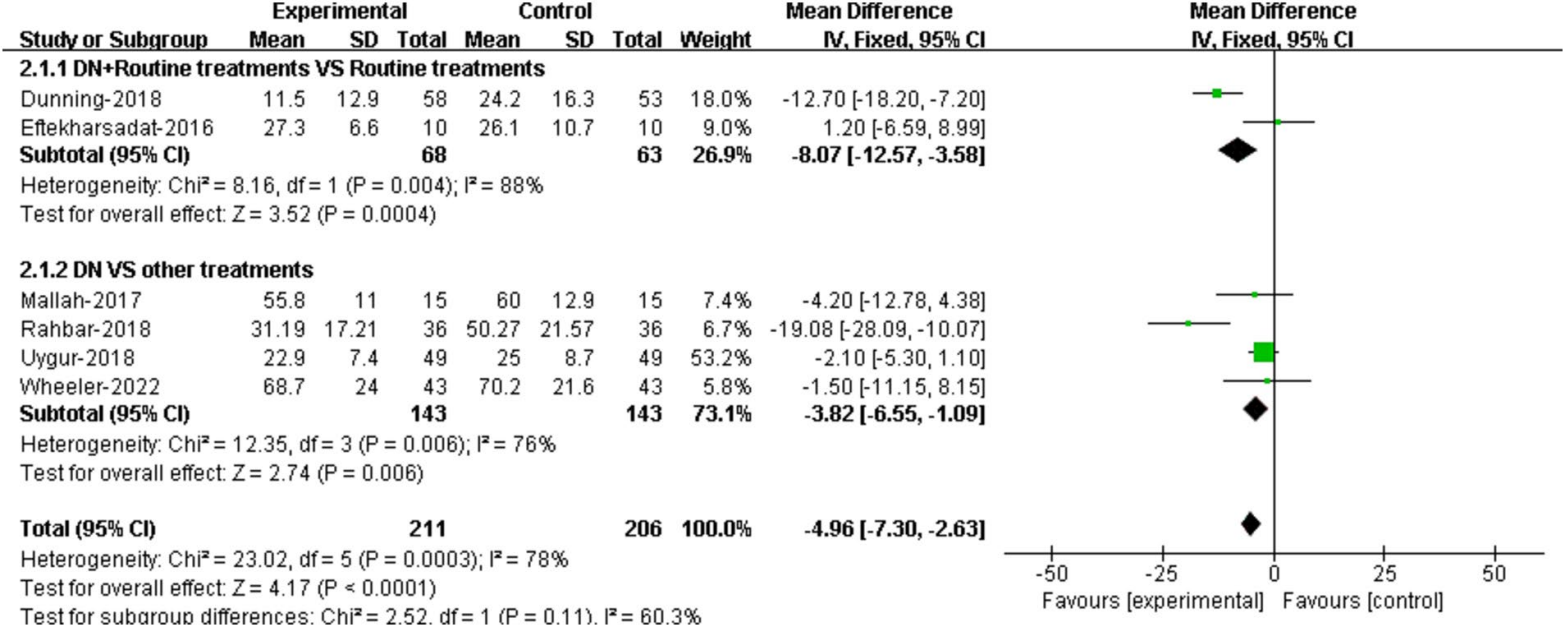
Forest plot illustrating the comparison of effectiveness on functional improvement between DN + routine treatments vs. routine treatments alone, and between DN vs. other treatments.

The comparisons of the effectiveness of DN + routine treatment vs. routine treatment alone on functional improvement were reported in 2 RCTs (22, 23). The results demonstrated that there was significant difference in FFI score between the two groups [95%CI (−12.57, −3.58), *p* = 0.004] (Figure 4).

The comparisons of the effectiveness of DN vs. other treatments on functional improvement were reported in 4 RCTs (24, 26, 28, 29). The results suggested that DN improved patients’ foot function compared to other treatments. The difference in FFI scores between two groups was statistically significant [95%CI (−6.55, −1.09), *p* = 0.006] (Figure 4).

### 3.5 Meta-analysis based on different assessment time

#### 3.5.1 Pain

**Figure 5:**
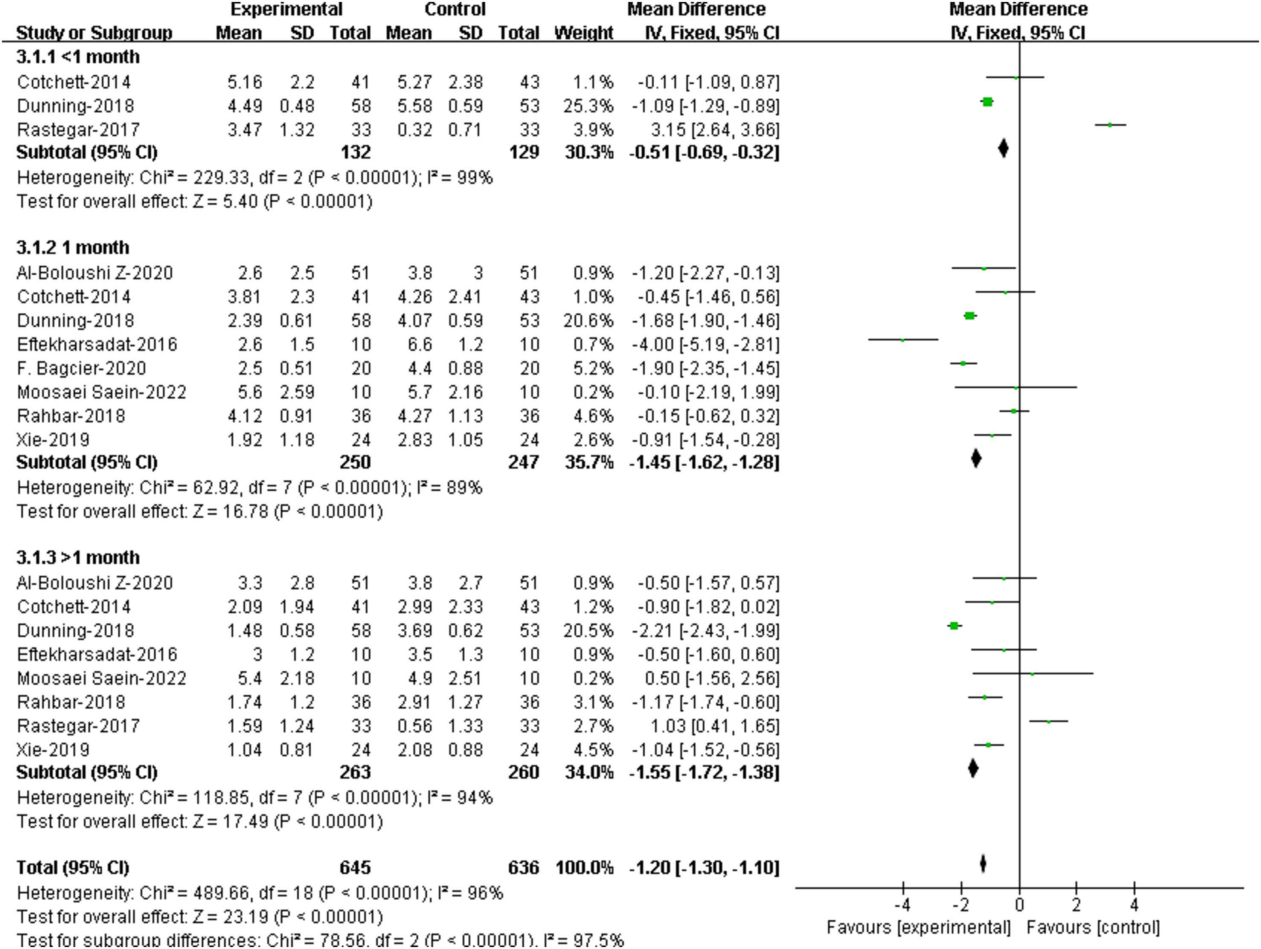
Forest plot illustrating the effectiveness of DN on pain at different assessment time.

3 RCTs provided assessment data of pain within 1 month. (21, 22, 27). Meta-analysis showed that there was significant difference in VAS/NPRS scores within 1 month between the two groups [95%CI (−0.69, −0.32), *p* < 0.00001] (Figure 5).

8 RCTs assessed pain at 1 month (19–23, 25, 26, 30) Results showed that patients receiving DN had lower VAS/NPRS scores at 1 month compared to that of the controls, and the difference were statistically significant [95%CI (−1.62, −1.28), *p* < 0.00001] (Figure 5).

8 RCTs assessed pain at over 1 month (19, 21–23, 25–27, 30). Results showed that patients receiving DN had lower VAS/NPRS scores at over 1 month compared to that of the controls, and the difference were statistically significant [95%CI (−1.72, −1.38), *p*<0.0001] (Figure 5).

#### 3.5.2 Functional improvement

**Figure 6:**
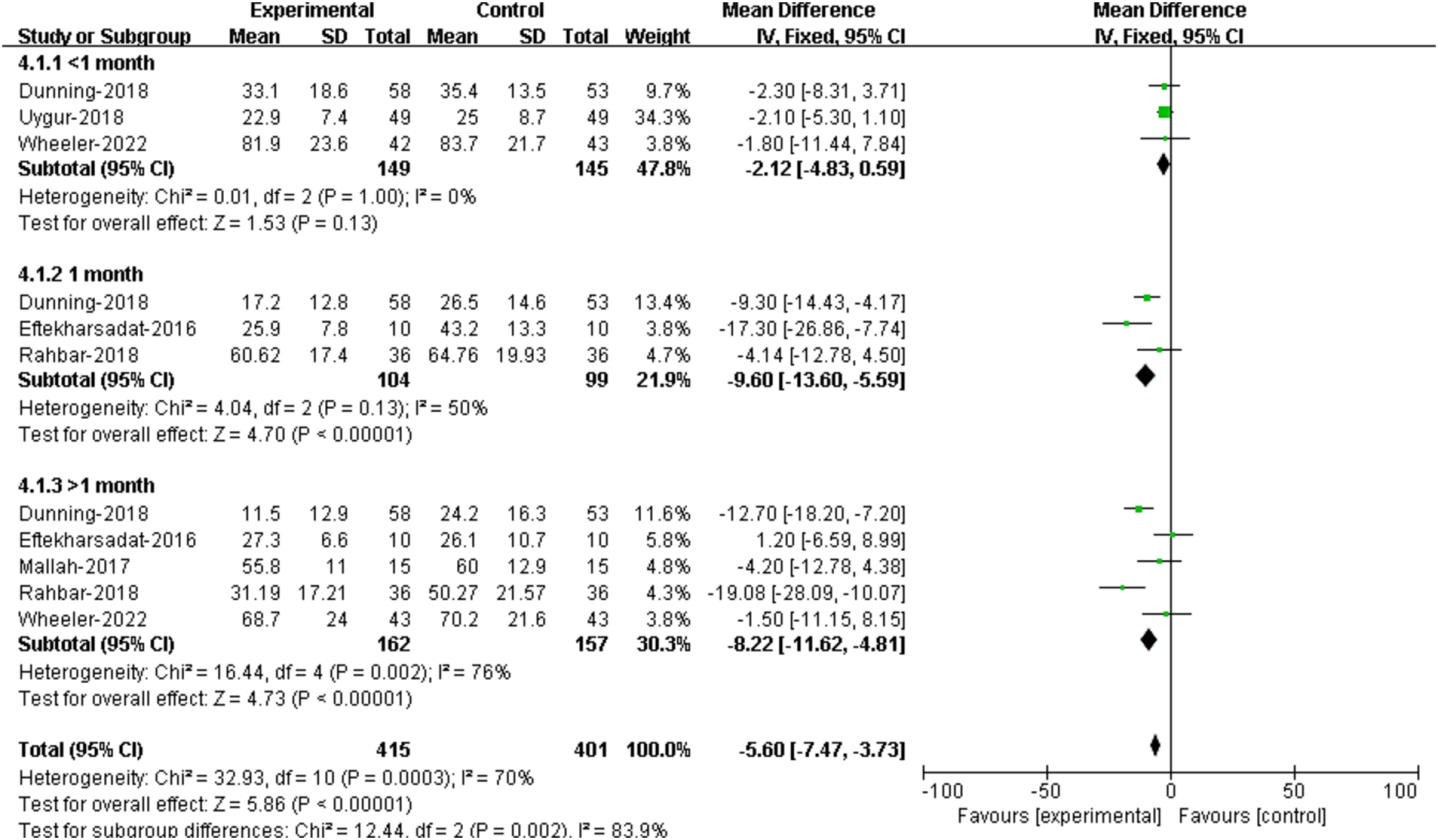
Forest plot illustrating the effectiveness of DN on functional improvement at different assessment time.

3 RCTs reported foot function assessment data within 1 month (22, 28, 29). Results showed that no significant difference was demonstrated between DN group and the control group [95%CI (−4.83, 0.59), *p* = 0.13] (Figure 6).

The comparison of treatment effectiveness on foot function at 1 month between DN and controls were conducted in 3 RCTs (22, 23, 26). The results suggested that patients receiving DN had lower FFI scores compared to that of the controls, and the difference was statistically significant [95%CI (−13.60, −5.59), *p* < 0.00001] (Figure 6).

5 RCTs provided assessment data of function improvement at over on month (22–24, 26, 29). The results showed that there was no significant difference in FFI scores at over 1 month between groups [95%CI (−11.62, −4.81), *p* < 0.00001] (Figure 6).

## 4 Discussion

It is recognized that DN is capable of reducing inflammation of the plantar fascia, enhancing local blood circulation, and relieving tissue internal pressure, resulting in extensive use of DN to treat PF clinically (20). The current study evaluated the effectiveness of DN on pain relieving and functional improvement in patients with PF. To gain a more precise and comprehensive understanding of its effectiveness, we conducted meta-analysis based on different control methods and different assessment time.

12 RCTs involving 781 participants with plantar fasciitis were analyzed in our study. In the analysis based on different control methods, the comparison of effectiveness on foot function between DN + routine treatments vs. routine treatments alone did not demonstrate significant difference, but the result favored the use of DN + routine treatments. In all the other comparisons (DN + routine treatments vs. routine treatments on pain), results suggested that patients who received DN had significant improvement in pain and foot function. These results were in line with the previous SRs. However, there was no significant difference between the DN group and the other treatment group.

In the analysis based on different assessment time, current evidence indicated that DN exhibited no obvious advantage over other treatments within 1 month. But at 1 month and at over 1 month, patients receiving DN had lower VAS/NPRS and FFI scores compared to that of the controls. These results suggested that DN relieved heel pain and improved foot function in a time-dependent manner. Possible reasons for the absence of effectiveness of DN within 1 month may be: (1) needling itself can cause pain in the heel, which may make confusion with the pain caused by the disease and thus interfering with the pain assessment (31). (2) DN has a cumulative effect in treating PF, the mechanical effects of the needle induce a remodeling of the collagen fibers of the plantar fascia that requires several weeks. (3) The repeated movements of the back and forward and rotations of the needle release the myofascial trigger points of the intrinsic muscles of the foot modulating their perfusion. (4) The dry needling of the posterior compartment of the leg modulates the tension of the fascial elements (especially the deep fascia located in between the muscles and the subcutaneous fat tissue) which are in the histological continuum with the plantar fascia (32). In other words, it takes time for DN to exert significant benefits on PF patients (33).

Even though our study supports the use of DN in treating PF, optimal management of PF requires other composite measures, including weight control (34), gait correction (35), and wearing appropriate shoes (36), to maintain the effects of DN and prevent disease recurrence. Adverse events, mainly including pain and bruise, were reported in 6 RCTs (50%). DN is generally safe given the fact that these adverse events were considered to be mild and easy to recover without the need of special care. However, in order to ensure patient safety, ultrasound-guided dry needling therapy may be considered in future studies to reduce local bleeding caused by accidental vascular rupture and avoid hematoma.

Our study has several strengths. Firstly, we have updated the literature by including 8 latest RCTs, all of which were published in the past 5 years, in our study. Secondly, we performed meta-analysis based on different control methods and assessment time, which may provide more precise information as to the effectiveness of DN. Nevertheless, there are limitations in this study. Most included RCTs have high risks of selective bias due to the lack of allocation concealment and blinding to patients. On the other hand, the included RCT articles were highly heterogeneous. Firstly, the control group intervention measures adopted in different studies are very different, with significant heterogeneity. The differences were great in the treatment sites, frequency, and operation methods of DN in the 12 RCTs. Secondly, using only clinical features to select the patients with “plantar fasciitis” some atypical pathologies such as the heel fat pad syndrome (37) and the disruption of the plantar fascia (38) may be included in the sample of fasciitis. Moreover, most RCTs have small sample size. All these limitations may influence the power of our conclusion.

Despite its limitations, this systematic review and meta-analysis provided moderate evidence supporting the use of dry needling to relieve pain and restore function in patients with plantar fasciitis. Furthermore, dry needling may take at least 1 month to take effects in patients with plantar fasciitis. More multi-center RCTs with high-quality, large sample size are needed to further conform our conclusion.

## Data Availability

The original contributions presented in the study are included in the article/supplementary material, further inquiries can be directed to the corresponding author/s.
